# Mercury source changes and food web shifts alter contamination signatures of predatory fish from Lake Michigan

**DOI:** 10.1073/pnas.1907484116

**Published:** 2019-11-04

**Authors:** Ryan F. Lepak, Joel C. Hoffman, Sarah E. Janssen, David P. Krabbenhoft, Jacob M. Ogorek, John F. DeWild, Michael T. Tate, Christopher L. Babiarz, Runsheng Yin, Elizabeth W. Murphy, Daniel R. Engstrom, James P. Hurley

**Affiliations:** ^a^Environmental Chemistry and Technology Program, University of Wisconsin–Madison, Madison, WI 53706;; ^b^US Geological Survey, Upper Midwest Water Science Center, USGS Mercury Research Laboratory, Middleton, WI 53562;; ^c^US Environmental Protection Agency (US EPA) Office of Research and Development, Center for Computational Toxicology and Exposure, Great Lakes Toxicology and Ecology Division, Duluth, MN 55804;; ^d^State Key Laboratory of Ore Deposit Geochemistry, Institute of Geochemistry, Chinese Academy of Sciences, Guanshanhu District, 550081 Guiyang, Guizhou, China;; ^e^Great Lakes National Program Office, US EPA, Chicago, IL 60604;; ^f^St. Croix Watershed Research Station, Science Museum of Minnesota, Marine on St. Croix, MN 55047;; ^g^Department of Civil and Environmental Engineering, University of Wisconsin–Madison, Madison, WI 53706;; ^h^University of Wisconsin Aquatic Sciences Center, Madison, WI 53706

**Keywords:** isotopes, invasive, fish, mercury, Lake Michigan

## Abstract

To understand the impact reduced mercury (Hg) loading and invasive species have had on methylmercury bioaccumulation in predator fish of Lake Michigan, we reconstructed bioaccumulation trends from a fish archive (1978 to 2012). By measuring fish Hg stable isotope ratios, we related temporal changes in Hg concentrations to varying Hg sources. Additionally, dietary tracers were necessary to identify food web influences. Through combined Hg, C, and N stable isotopic analyses, we were able to differentiate between a shift in Hg sources to fish and periods when energetic transitions (from dreissenid mussels) led to the assimilation of contrasting Hg pools (2000 to present). In the late 1980s, lake trout δ^202^Hg increased (0.4‰) from regulatory reductions in regional Hg emissions. After 2000, C and N isotopes ratios revealed altered food web pathways, resulting in a benthic energetic shift and changes to Hg bioaccumulation. Continued increases in δ^202^Hg indicate fish are responding to several United States mercury emission mitigation strategies that were initiated circa 1990 and continued through the 2011 promulgation of the Mercury and Air Toxics Standards rule. Unlike archives of sediments, this fish archive tracks Hg sources susceptible to bioaccumulation in Great Lakes fisheries. Analysis reveals that trends in fish Hg concentrations can be substantially affected by shifts in trophic structure and dietary preferences initiated by invasive species in the Great Lakes. This does not diminish the benefits of declining emissions over this period, as fish Hg concentrations would have been higher without these actions.

## Significance

Elevated mercury in fish poses risks to fish-consuming wildlife and humans. Tracing sources of mercury by analyzing stable isotope ratios leads to improved source-receptor understanding and natural resource management. This work utilizes fish and sediment archives to trace the response to recent domestic mercury mitigation actions. Fish and sediments rapidly responded to a source perturbation contemporaneous with the reduction of mercury in the late 1980s. Subsequently, energetic pathways were altered due to dreissenid invasions, which dampened the expected decrease in fish mercury concentration. These findings reveal the importance of domestic mercury sources relative to global mercury to the Great Lakes. Results also show methylmercury concentrations in fish are sensitive to changes in trophic structure and diet driven by invasive species.

Mercury (Hg) is ubiquitous naturally, but since the mid-1800s anthropogenic activity has increased atmospheric concentrations by 3 to 4 times, enriching Hg reservoirs worldwide (1, 2). Gaseous elemental Hg emitted to the atmosphere has a long atmospheric residence time (about 6 to 12 mo), resulting in deposition and contamination in even the most remote areas (1). While in the atmosphere, reactions with oxidants produce highly water soluble, divalent Hg that is susceptible to rapid deposition to aquatic ecosystems, and subsequent microbial conversion to methylmercury (MeHg), a highly bioavailable neurotoxin (1, 3). Bioaccumulation of MeHg results in fish concentrations over a million times greater than surrounding waters, which can lead to detrimental effects to fish and to humans and wildlife (4).

Fish contaminant monitoring in the Great Lakes began by the mid-1970s for lake trout (*Salvelinus namaycush*). By the late 1970s, the US Environmental Protection Agency (US EPA) Great Lakes Fish Monitoring and Surveillance Program (GLFMSP) was established to assess ecosystem health using top predator fish, which were then archived as sentinels for monitoring chemical contaminants. The GLFMSP archive offers the opportunity to assess long-term trends in Hg bioaccumulation. This rare sample set allowed us to examine how variations in ecosystem characteristics and changes in regulatory actions have affected fish Hg bioaccumulation. Multiple factors affect total Hg (HgT) concentration in fish, including: changes to Hg loading and cycling (HgT inputs, methylation rates of Hg, uptake of MeHg by primary producers); photochemical demethylation rate of MeHg, fish bioenergetics, and diet (changes in fish metabolism or growth rate, spawning, changes in trophic position or foraging habitat, varied fish size or age); and ecosystem chemical characteristics (pH and dissolved organic carbon [DOC] content) (4–9). Fish archives can be powerful indicators of change, but multidecadal archives are rare (10), highlighting the tremendous value of analyses associated with these biomonitoring efforts.

Nationally, several regulations have been implemented since the early 1980s that affect Hg use, releases, and loading to the Great Lakes region. These include the US Clean Air Act, Mercury Export Ban Act of 2008, SO_x_ and NO_x_ pollution controls, and the 2011 promulgation of the Mercury and Air Toxics Standards (MATS) rule, with required compliance in 2015 (11). In addition to these mitigation strategies, changes in energy production, namely the conversion from coal to natural gas, have resulted in further decreases in Hg emissions (11). These domestic mitigation strategies are important to reducing the contributions of regional Hg sources to the Great Lakes (12). Hg emission sources vary in the species of Hg released [Hg^0^, Hg(II), and Hg_p_]; therefore, while reduction of all Hg is essential to reducing ecosystem loads, the elimination of sources containing high proportions of Hg(II), such as incineration-sourced Hg, provides immediate local responses in aquatic ecosystems (12). Considering differences in Hg reactivity of varied Hg species is important to understanding the potential of source Hg to become methylated and subsequently bioaccumulate in fish (3, 6). Understanding Hg speciation and the processes that affect MeHg formation and bioaccumulation is crucial to assessing multiple drivers of trends in fish Hg concentrations (1, 3, 12).

Tracing historical Hg deposition and the success of Hg mitigation strategies typically invoke the assessment of inorganic Hg reservoirs [peat (13), ice (14), sediment (15), and soil cores (16)]. Remote regions of the world have served as interpretable archives for tracing global signals. The degree of anthropogenic enrichment can be determined by comparing these regions to areas experiencing greater anthropogenic influence. These historical trend investigations are useful for reconstructing loading and can provide insights to help quantify Hg inputs to ecosystems. However, these media typically integrate all of the Hg sources to a receiving water body or ecosystem. Thus, they are not readily used to assess discrete Hg sources. In addition, different Hg sources possess varying potential for methylation and bioaccumulation. As a result, as others have recently noted (17), paleo-reconstruction of Hg inventories cannot directly assess exposure routes of MeHg to fish.

By coupling measurements of stable isotope ratios (C, N, and Hg) on tissues from this long-term fish tissue archive, we can better resolve the bioavailability of certain Hg sources, the susceptibility of Hg sources to methylation, and changes in fish diet habits. Hg stable isotopic fractionation has been used to identify inorganic Hg sources to ecosystems and to understand in situ processes occurring during transport (5, 13, 16, 18, 19). The large range in natural mass-dependent fractionation of Hg (MDF, denoted as δ^202^Hg) is a result of kinetic and equilibrium reactions (6) that can be divided into reactant-favored (−δ^202^Hg) or product-favored (+δ^202^Hg) reservoirs. MDF is common in most Hg reactions, including those relevant in the environment: adsorption, photochemical reduction, photochemical demethylation of MeHg, and microbial methylation and demethylation (5). In contrast, mass-independent fractionation (MIF, denoted as Δ^199^Hg or Δ^200^Hg) is a phenomenon not commonly observed in heavy metals (5). Hg is susceptible to multiple odd-MIF processes and at least 1 even-MIF process, that together result in the potential for multidimensional tracking of Hg sources and transformations (5, 13, 16, 18, 19). In aquatic ecosystems, odd-MIF is typically the result of photochemical reduction of inorganic Hg (measured in sediments, particulates, and water) and photochemical demethylation of MeHg (measured in biota) (5). In fish, odd-MIF tracks the extent of photochemical demethylation, typically linked to water clarity and, in some instances, the source MeHg (20–23). Empirically, even-MIF serves as a binary tool for determining the relative importance of atmospherically sourced Hg (13, 23, 24), with positive reservoirs reflecting precipitation (+Δ^200^Hg, oxidant product) (25, 26) and negative reservoirs reflecting gaseous elemental (−Δ^200^Hg, reactant) (19) influence.

C and N stable isotope ratios are used to trace food web pathways, including identifying the energy source and estimating trophic position (27–29). In the Upper Great Lakes, nearshore carbon sources, such as benthic algae and littoral vegetation, are ^13^C-enriched compared to open-water phytoplankton, because of habitat-specific differences in both the isotopic composition of dissolved inorganic carbon pools and fractionation during carbon fixation (30–32). In addition, food webs demonstrate ^15^N enrichment with depth, which is presumably caused by microbial processing of sinking organic matter (33, 34). In Lake Michigan, there is additional complexity to consider because the offshore pelagic food web demonstrates ^15^N enrichment compared to the nearshore food web (28, 30). Furthermore, δ^15^N values of organisms systematically increase with trophic position and thus can be used to determine effective trophic position (29, 30). While trophic position is typically useful in tracing the efficiency of contaminant accumulation in fish (29), here we focus on the relative δ^15^N values along short timescales (5 to 10 y). By assuming a constant baseline, we can then infer changes to δ^15^N in fish are the result of changed energy pathways rather than changed trophic position. When paired together, C and N stable isotope ratios can serve as powerful tools to trace dietary shifts and habitat-specific energy pathways in Great Lakes fishes (28, 30).

Lake Michigan has undergone substantial changes in contaminant loading since the 1970s and in food web shifts following the dreissenid mussel invasion of the 1990s (35–37). Therefore, the GLFMSP lake trout archive represents an excellent opportunity to explore the effect these changes have had on MeHg sources and bioaccumulation to top predator fish. In addition, because of the Lake Michigan Mass Balance study and subsequent studies (38–41), the lake has been the subject of pioneering Hg research within the Great Lakes; however, consistently monitored long-term temporal data for Hg in Lake Michigan fish are scant compared to other Great Lakes (42), which are served by both United States and Canadian monitoring programs. Based on our prior work in the Great Lakes (18, 23), we expect that, due to low sedimentary MeHg fluxes and watershed loading, sediment Hg concentrations in Lake Michigan are not well corroborated with MeHg concentrations in fish. We also expect that reductions in regional Hg emissions will be reflected in Hg isotope ratios in fish and sediments (6). Thus, as domestic Hg mitigation strategies have affected the emission portfolio of Lake Michigan’s airshed during the time covered by the archive (1978 to 2012), we hypothesize that changes in the Hg isotopic composition and MeHg concentration of fish will be evident. Second, we expect that the lake-wide food web response from dreissenid invasions will be reflected in MeHg isotope signatures in fish due to increased water clarity and lake trout diet shifts. Here we couple stable isotope analyses of C, N, and Hg to better understand the impact reduced Hg emissions and food web shifts exert on Hg bioaccumulation in a key Lake Michigan biomonitor.

## Results and Discussion

### HgT Concentration and Energy Sources Using Traditional Stable Isotope Ratios.

**Fig. 1.**
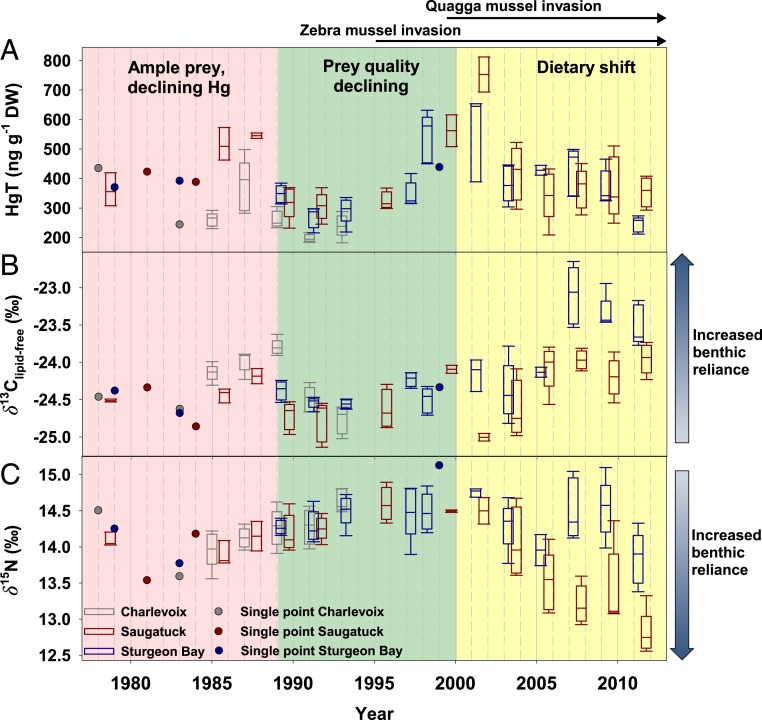
Tracing the influence of varying perturbations to Lake Michigan that affected HgT concentration in lake trout composites (*n* = 132) (*A*), and energetic pathways, traced by lipid-corrected δ^13^C (*B*) and δ^15^N (*C*) from 1978 to 2013. Each data point represents a composite of 5 lake trout, and the boxplots were used when 2 to 5 separate composites were measured within a single year. Box plots indicate the mean and quartiles of the fish composites sampled in a site and year. Whiskers represent the 10th and 90th percentiles. Plot color indicates site, with gray, red, and blue representing Charlevoix, Saugatuck, and Sturgeon Bay, respectively. Locations of these sites may be found in *SI Appendix*, Fig. S3. Three considerable perturbations to lake trout and Hg cycling are marked: major Hg source shifts due mitigation strategies, invasion of zebra mussels, and quagga mussel invasions. The background highlights the 3 time-dependent subsections discussed in the text.

Fish composites, each composed of five 400- to 600-mm whole-body lake trout grinds collected during the fall season from 1978 to 2012, averaged 361 ng g^−1^ HgT dry weight (117 ng g^−1^ 1 SD; *n* = 132 composites, 660 individual fish) with a maximum HgT of 812-ng g^−1^ dry weight and a minimum 182 ng g^−1^ (Fig. 1*A*). No composites exceeded the 300 ng g^−1^ whole-body, wet weight (composites averaged 75% water, ∼1,200 ng g^−1^ dry weight) lowest observed effect residue for fish health (43). Fig. 1 identifies key subsections of our time series that align well with known lake trout food source inflections: Ample prey with generally declining Hg emissions (1978 to 1989) (Fig. 1, red), prey quality declining due to declines in *Diporeia* spp. (1989 to 2000) (Fig. 1, green), and dietary shifts as round goby (*Neogobius melanostomus*) becomes an increasingly more prominent dietary item (2000 to 2012) (Fig. 1, yellow). From 1978 to 1989, HgT concentrations were somewhat variable but continually decreased until stable from 1989 to 1993 (Pearson’s ρ = −0.42). From 1993 to 2001, HgT concentrations increased by ∼400 ng g^−1^ (ρ = 0.80). From 2001 to 2012, concentrations decreased by ∼300 ng g^−1^. The decrease in Hg loading to Lake Michigan, corroborated by sediment cores (*SI Appendix*, Fig. S1) (15) is in direct conflict with fish Hg trends following 1995. To better understand the decoupled Hg patterns between fish and sediments then, an understanding of the importance of fish diet or tracing of energy pathways is necessary.

Biological and aquatic chemical factors may also affect Hg concentrations in fish. Reproductive cycles, for example, can affect HgT concentrations; however, our composites are, on average, equally composed of males and females, and were continually collected during the spawning period. Thus, variations in HgT concentrations, are not attributable to gender or reproduction cycles. Through previous work, we also conclude that DOC concentrations have been stable, while pH slightly increased following the early 1990s and slowly declined since the early 2000s (44). We therefore do not believe these to be a major driving factors to observed trends. Here, we focused our research on the effects on fish Hg concentrations due to diet-related factors (using C and N stable isotope ratios), changes to Hg loading, water clarity, and sources of inorganic Hg.

Invasive zebra (*Dreissena polymorpha*) and quagga mussels (*Dreissena rostriformis bugensis*), which arrived in the early 1990s and 2000s, respectively, dramatically changed carbon and nutrient dynamics in Lake Michigan by efficiently filtering phytoplankton and terrigenous inputs and rerouting energy and nutrients into nearshore and benthic habitats (45). The collapse of *Diporeia* spp. (a benthic amphipod) populations during the onset of mussel invasions led to changes in dietary strategies of alewife (*Alosa pseudoharengus*) and the energy pathways therein (37, 46, 47) (Fig. 1, 1989 to 2000). This, in combination with dense piscivore populations, ultimately led to a substantial decrease in prey fish (e.g., alewife), forcing lake trout to transition a proportion of their dietary habits (Fig. 1, 2000 to 2012) (48). During this same period, round goby, an invasive benthic fish that consumes dreissenid mussels, became prominent in the Lake Michigan food web (48). Alewife has remained the most important component of lake trout diet (>50%), but the contribution of round goby has increased through the late 2000s, although it remains relatively small (<30%) (37, 46, 49, 50). Because of reduced quality in prey, the growth rate of lake trout in Lake Michigan has slowed (51, 52). To confirm whether invasive species have resulted in dietary shifts in lake trout, we utilized lipid-normalized δ^13^C (δ^13^C_lipid-free_) and δ^15^N values (28).

The δ^13^C_lipid-free_ values in nearly all of the fish composites sampled during 1978 to 2000 were similar, within ∼2.50‰ (−24.23 ± 0.48; *n* = 77) (Fig. 1*B*). After 2000, δ^13^C_lipid-free_ values became distinct between Sturgeon Bay and Saugatuck, and δ^13^C_lipid-free_ steadily increased through time (0.08 to 0.11‰ y^−1^). This change was coincident with the dreissenid mussel invasion, increases in Secchi depth (53), and increased nearshore primary production, which is a zone of increased MeHg enhancement when compared to offshore regions (28). In the Great Lakes, benthic algae contribute to the nearshore food web and are substantially ^13^C-enriched relative to phytoplankton; thus, benthic, nearshore fishes have higher δ^13^C values than offshore fishes (32). Similarly, during particulate organic matter sedimentation, microbial processing enriches its ^13^C content, resulting in slightly higher δ^13^C values in benthic consumers than pelagic consumers (33, 54). The shift in lake trout δ^13^C_lipid-free_ values indicated a corresponding energetic shift toward either the benthos (28) or the nearshore environment, or some combination thereof, following the dreissenid mussel invasion (47). In many of the Great Lakes, the δ^13^C of organic matter in sinking particles was conserved through sedimentation and burial (33, 54). It was therefore plausible to reconstruct δ^13^C baselines using sediment cores. Since the 1970s, negative δ^13^C baseline shifts have been observed (roughly 1‰) (33) likely attributable to decreased offshore productivity. We therefore concluded that the ^13^C-enrichment in lake trout was not the result of an underlying shift in the baseline δ^13^C because the shifts in regional sediment δ^13^C values and Lake Michigan lake trout δ^13^C_lipid-free_ values were opposite in direction.

For fish collected between 1978 and 2000, δ^15^N values spanned a larger range relative to δ^13^C_lipid-free_ values and continually increased through time (0.04‰ y^−1^; ρ = 0.51) (Fig. 1*C*), resulting in a net 0.8‰ increase from 1978 to 2000 (mean ± SD = 14.30 ± 0.31; *n* = 77). While alewife remained the mainstay of lake trout diet throughout this study time (49, 55–57), this shift was likely due to decreasing alewife density through the 1980s and early 1990s, and lake trout targeting alternative ^15^N-enriched benthic prey with similar δ^13^C values, such as bloater (*Coregonus hoyi*; until the early 1990s) (28, 48).

Following 2000, a rapid decrease in lake trout δ^15^N values occurred at Saugatuck and Sturgeon Bay (−0.14 and −0.05‰ y^−1^, respectively). This trend continued through 2010 at Saugatuck, whereas after 2006, δ^15^N values at Sturgeon Bay returned to values like the 1990s. While these responses differ somewhat, the net change in lake trout δ^13^C_lipid-free_ (enriched) and δ^15^N values indicated increased reliance on the benthic food web. At the base of both the pelagic and benthic food web pathways in Lake Michigan, offshore pelagic δ^15^N values were higher than nearshore δ^15^N values (28). The change in lake trout δ^15^N values between the 2 stations was large and the result of ^15^N depletion in nearshore (Saugatuck, 61-m depth) regions because of altered N cycling by dreissenid mussels, favoring nitrification from the nearshore shunt and changed lake trout dietary pathways toward dreissenid mussels (37, 45). In contrast, in offshore waters (Sturgeon Bay, 119-m depth), benthic nitrogen is largely from the atmosphere, biologically fixed (34), and delivered seasonally during turnover. Therefore, offshore isotopic baseline change is slower and smaller than in the nearshore, given that fluxes of new nitrogen are small relative to the total nitrogen budget. The difference in δ^15^N values after 2005 between Saugatuck and Sturgeon Bay may also reflect lake-wide changes in the δ^15^N baseline because ^15^N depletion was observed in the northern basin sediments relative to the southern basin during the same period, albeit of lesser magnitude than observed in lake trout (roughly 0.5 to 1‰) (33, 54). Caution is necessary, however, as sediment cores provide less diagnostic information when compared to the δ^13^C value of organic matter because δ^15^N values in sediment are affected by overlying water column productivity as well as in situ nitrification and denitrification (33, 54).

### Using Δ^199^Hg as a Tracer for Water Quality and MeHg Photochemical Processing.

**Fig. 2.**
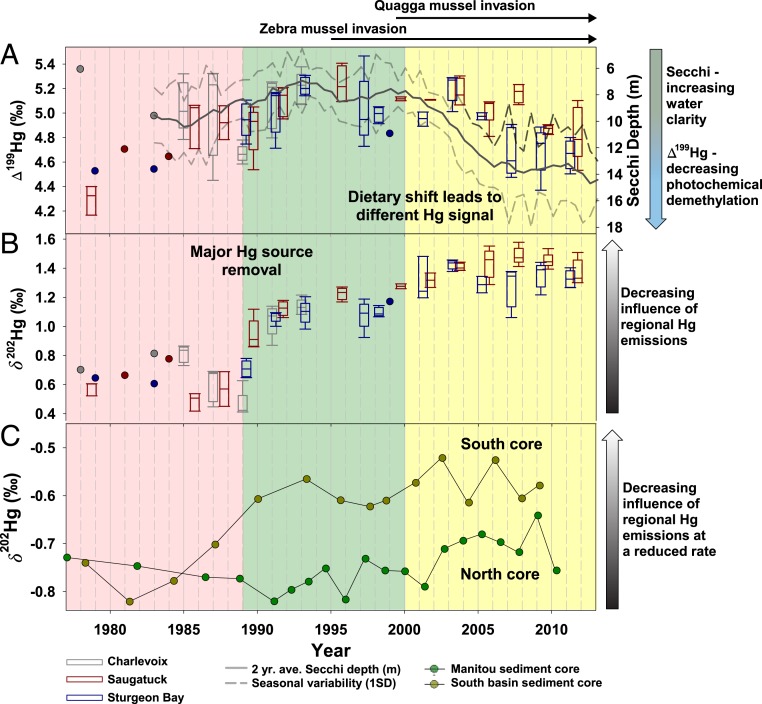
Tracing Hg isotope composition in fish and sediments. (*A*) Δ^199^Hg and (*B*) δ^202^Hg in Lake Michigan lake trout composites (box plots, left axis); (*C*) δ^202^Hg in 2 Lake Michigan sediment cores. Secchi depth (*A*) was collected at a broad range of sites within a given year and data were retrieved from the US EPA data exchange database (https://cdx.epa.gov). This centerline of the Secchi depth represents a 2-y average of all sites and the dotted lines, represent the spring and summer variability (1 SD). Detailed description of the fish data may be found in Fig. 1. Core data points represent a single sediment slice at the mid-interval year. Marked on the figure top are 3 considerable perturbations to lake trout and Hg cycling, a major Hg source shift from mitigation strategies, and the zebra and then quagga mussel invasions. The background highlights the time-dependent subsections discussion in the text.

From 1978 to 1995, Δ^199^Hg values averaged 4.95 ± 0.33‰ (*n* = 61) (Fig. 2*A*) and increased slightly through this period. Following 1995, Δ^199^Hg values slowly declined (∼0.02‰ y^−1^) until 2007, after which values became steady. Previously, Δ^199^Hg has been considered a positive predictor for overall water clarity, determinable by DOC content, Secchi depth (53), and direct light attenuation profiles over a diverse set of waterbodies (23, 58). Regular measurements of Secchi depth became available for Lake Michigan following 1983 (Fig. 2*A*, gray lines) and can be found at the US EPA Great Lakes National Program Office, Great Lakes Environmental Database (https://cdx.epa.gov). Beginning in 1983, Secchi depth decreased slightly until achieving a water clarity minimum (7 m) in 1993. After 2000, water clarity rapidly increased, likely a result of dreissenid mussel invasion (45). During this period, Δ^199^Hg values decreased slightly. Previously, we made observations between Secchi depth and Δ^199^Hg in predator fish of the Great Lakes (23) that led us to expect that a large increase in water clarity following quagga mussel invasion would to lead to a detectable increase in lake trout Δ^199^Hg values. While it is reasonable to assume we could predict changes in Δ^199^Hg values with enhanced water clarity, food web pathways (i.e., benthic or pelagic energy sources, and trophic position) influence Δ^199^Hg values more than previously recognized (22, 23, 58). After 2000, lake trout reliance on benthic dietary pathways increased due to changed dietary pathways for alewives and increased consumption of round goby. This resulted in the bioaccumulation of Hg that was less photochemically fractionated (Δ^199^Hg) in comparison to pelagic Hg sources (23, 58). Simultaneously, both lake trout condition—as indicated by lipid content (*SI Appendix*, Fig. S2)—and growth rates declined (51, 52). Due to slower growth, as previously noted, lake trout Hg concentration after 2000 was elevated relative to the early 1990s (51, 52).

### δ^202^Hg as a Source Indicator.

δ^202^Hg has been used as a surrogate for sources of inorganic Hg because different Hg reservoirs exhibit distinct δ^202^Hg ranges (5). In cores of sediment and peat, δ^202^Hg is used as a source indicator to investigate historical deposition or changes in Hg source profiles (13, 15). In fish, however, using δ^202^Hg for MeHg in a diagnostic manner is more challenging than simply identifying inorganic Hg sources, because MDF also occurs during photochemical demethylation (5), during microbial methylation and demethylation (59), and potentially during metabolic processing (22, 60). We expect, however, that these variables will not impart considerable changes to the δ^202^Hg of these lake trout due to the consistency in sampling protocol.

Lake trout δ^202^Hg values from 1978 to 1988 were similar (0.64 ± 0.15‰, *n* = 21) (Fig. 2*B*); however, from 1988 to 1996, δ^202^Hg values increased (∼0.6‰; ρ = 0.81) rapidly for all sites to ∼1.2‰. Measurements of δ^15^N and δ^13^C_lipid_ _free_ provided evidence that this δ^202^Hg shift was not due to an altered dietary pathway (e.g., benthic versus pelagic) but rather from a change in Hg source that acted independently upon δ^202^Hg, resulting in a positive MDF shift. The coherent response of all 6- to 8-y-old fish (52) suggested a large change in source, such as a cessation of a δ^202^Hg-deplete point source.

Understanding the response of δ^202^Hg in fish requires knowledge of whole-body isotopic turnover rates of C, N, and Hg in fish. Isotopic turnover of C and N was estimated to be 6 mo to 2 y in adult fish (61), with 600- to 700-mm lake trout reaching turnover in about 1.2 y (∼2.7 kg whole-body weight) (62). This biennial sampling therefore captured time-discrete δ^13^C and δ^15^N in lake trout, and results can trace the sensitive dynamic equilibrium sufficiently. Other studies have demonstrated that MeHg in fish has a modestly longer half-life than C and N (60, 62) and that fish can respond rapidly to MeHg source perturbations (6). Based on those observations, along with the rapid, coherent δ^202^Hg shift in lake trout, we believe that the lake trout rapidly respond to cessation in Hg emissions from regional sources. Similarly, we predict that only atmospherically transported sources would uniformly deposit to Lake Michigan; therefore, a cessation of Hg would result in uniform response.

During the time this fish archive spans (1978 to 2012), several Federal environmental regulations were implemented that resulted in substantial reductions in United States atmospheric Hg emissions. It is important to note that most of these actions did not necessarily target Hg-emission reductions, but nonetheless reduced Hg emissions were realized as a secondary consequence. For example, 1990 amendments to the Clean Air Act were intended to reduce many toxic chemical emissions from medical and municipal waste incineration, including SO_x_ and NO_x_ acidic gases, as well particulate matter <2.5 micrometers in diameter (PM_2.5_). While these actions were not specifically intended to reduce Hg emissions, removing primary targets resulted in secondary benefits (e.g., Hg reductions with decreasing high sulfur coal use). Phasing out Hg use in battery manufacturing in 1996 (Mercury-Containing and Rechargeable Battery Management Act) more directly reduced Hg emissions by eliminating these products in waste streams intended for incineration. Collectively, these actions represented the largest reduction in domestic atmospheric Hg emissions over the period studied (63), and thus are likely responsible for the observed increase in δ^202^Hg from 1988 to 1996 (Fig. 2*B*). These actions also most efficiently targeted particulate bound Hg and Hg(II), both of which rapidly deposited to the lakes (6, 12). Furthermore, improved NO_x_ (selective catalytic reduction) and SO_x_ control strategies (Clean Air Interstate Rule, mid-2000s) resulted in additional Hg removal (11). The Mercury Export Ban of 2008 further decreased the supply-chain availability of Hg in manufacturing globally, thereby reducing global emissions. Finally, the MATS rule was promulgated in 2011 and placed requirements on Hg emission reductions by the largest remaining source at that time, electric power generation. At the same time the MATS rule was implemented, substantial increases in natural gas availability resulted in shift in many electric power generation units, from coal to natural gas (coal use for electrical generation in the Midwest United States declined 30 to 40% in the 2000s; www.eia.gov/coal) which has also unintentionally reduced regional emissions of Hg (11). In total, North American Hg emissions declined by a factor of 3.8 from 1990 to 2010 (469 to 124 Mg y^−1^) (11). Taken together, this history of emission declines reduced gaseous elemental and divalent Hg concentrations in the airshed of Lake Michigan (11, 63) and atmospheric deposition of Hg in the Great Lakes region (64). From these reduced Hg emissions, we postulate that corresponding shifts in the isotopic source portfolio of Hg has likewise arisen and was observed in our measured lake trout δ^202^Hg values. More specifically, we hypothesize the reduction of Hg from various incineration processes has resulted in a net enrichment (+δ^202^Hg) of the Hg isotopic signature in the atmosphere as observed previously (65, 66).

Following 2000, Hg isotope transitions were decoupled from the expected shifts associated with changes to photochemical demethylation (20, 23, 58). Lake trout Δ^199^Hg values decreased following 2000 (Fig. 2*A*), indicating water clarity was not the driver for increasing δ^202^Hg values. Instead, a change to increased reliance on benthic food web pathways [defined by some combination of increased reliance on round gobies (50) or changed dietary strategies of prey fish due to collapsed *Diporeia* (46, 47)], as indicated by C and N stable isotope ratios (Fig. 1 *B* and *C*), resulted in the bioaccumulation of a different MeHg source. Furthermore, δ^13^C and δ^15^N values were dissimilar between the northern (Sturgeon Bay) and southern (Saugatuck) sites, indicating the fish were not routinely mixing between the sites over a time scale equal to their isotopic turnover (1 to 2 y) (61). As such, similarity in Hg isotopic compositions between the 2 sites reaffirmed our finding that the MeHg source shift was not related to regional point sources (for example, rivers or discharges), but was most likely a diffuse source, such as the atmosphere. Only with the combination of −δ^15^N, +δ^13^C_lipid_ _free_, −Δ^199^Hg, and +δ^202^Hg values during the 2000s and by including lake trout harvested in the north and south Lake Michigan, could we hypothesize an ecosystem-wide benthic transition in both regions. This finding was also corroborated by the parallel shift in −δ^15^N and +δ^13^C_lipid_ _free_ observed in the Lake Michigan invertivore lake whitefish (*Coregonus clupeaformis*) (67) following dreissenid mussel invasion.

### Comparing a Fish Archive to Sedimentary Accumulation.

Because of their excellent capacity to reconstruct Hg deposition trends, sedimentary archives have, at times, been used as a proxy to assess Hg loading to fish and to trace Hg deposition fluxes and sources to sediment through time (2, 13, 15). In regions with direct Hg contamination, the Hg source is usually sequestered in sediments, where it is available for methylation, and it is in these settings that inferences between sediment and fish can be drawn. However, in aquatic ecosystems with complex source portfolios and in which MeHg fluxes to overlying waters from sediments are a minimal contributor, sedimentary archives may be insufficient to predict MeHg concentrations in fish. In Lake Michigan, we previously hypothesized that MeHg was likely produced in the water column (23, 35). It was therefore plausible that Hg sources depositing to the near surface lake environment were more likely to be bioaccumulated fish than Hg sources that reach the sediments, which reflect a combination of both remnant Hg from surface deposition and particulates carried by lake currents (18).

Here we found that the magnitude of the sediment δ^202^Hg response was subdued when compared to the fish response around 1990, supporting our hypothesis that fish and sediment signals were tracing different Hg sources or pathways (Fig. 2*C*). We examined 2 sediment cores, sliced to 2- to 3-y resolution, from Lake Michigan near the sites of fish collection (*SI Appendix*, Fig. S3 and Table S1). We observed that the δ^202^Hg response in sedimentary Hg was only about 30% (0.1 to 0.2‰) (Fig. 2*C*) that of the fish. Furthermore, the absolute ranges of Δ^199^Hg and δ^202^Hg values were dissimilar between the cores and fish because sediments reflected inorganic Hg that was not extensively photochemically reacted. Sediment Δ^199^Hg values for the north Manitou and southern basin cores (*SI Appendix*, Table S2) were relatively constant at 0.21 ± 0.02‰ (*n* = 21) and 0.08 ± 0.02‰ (*n* = 17), respectively. Neither core location appeared to convincingly respond to the increase in water clarity, which would have been expected to produce a change in Δ^199^Hg, indicating that sources of sedimentary Hg were not closely linked to shifts in productivity in Lake Michigan.

Researchers often compare the slope of photochemical demethylation of MeHg (Δ^199^Hg:δ^202^Hg slope = 2.4) between fish (containing MeHg) and sediments (dominantly composed of inorganic Hg) within an ecosystem to draw inferences about sources of Hg to fish (5, 22). This relationship is valuable when sediment MeHg fluxes are elevated, for example in hypolimnion of anoxic lakes and in riverine systems (5, 22, 68). Furthermore, variations in the relative degree of microbial methylation and demethylation can affect the Hg isotope ratios of MeHg fluxes from sediments (3, 59). Here the combined dated sediment profile and lake trout archive in Lake Michigan allowed us to investigate paired sediment and fish Hg isotopic composition over time. During this time, we assumed Hg methylation and demethylation rates in sediments have remained constant. The sediment to fish Δ^199^Hg:δ^202^Hg relationship began with a slope of 3.5 in 1978 (*SI Appendix*, Fig. S4) and decreased in a linear fashion (*R*^2^ = 0.65) to 2.4 in 2013 at a rate (m = 0.035) of 1 to 1.5% per year (change in Δ^199^Hg:δ^202^Hg = −0.04 y^−1^). Wet deposition of Hg, has been decreasing at a rate similar to this Δ^199^Hg:δ^202^Hg slope change (−1.6 ± 0.3% y^−1^) in North America from 1990 to 2013 (11) and, for this reason, we propose that in Lake Michigan, the Δ^199^Hg:δ^202^Hg slope actually traces the signal of incoming precipitation, which is then methylated in the water column (23, 69) rather than as a sedimentary MeHg efflux. We would, however, need temporally similar precipitation samples to confirm this hypothesis. We postulate that the Δ^199^Hg:δ^202^Hg slope of 2.4 measured between fish and sediment in the 2010s was the result of profundal sediments reflecting a signal from recently deposited algal and detrital remains from the water column. In Lake Michigan however, this has not always been the case because, historically, fish received a proportionally greater amount of atmospherically delivered Hg, a subtlety that would be missed without the paired dated sediment and fish collections.

### Δ^200^Hg Comparison between Fish and Sediment Cores over Time.

Increasingly, Δ^200^Hg has been used as a tracer for both gaseous elemental (−Δ^200^Hg) and oxidized atmospheric Hg (+Δ^200^Hg − precipitation) (70). Δ^200^Hg is thought to form in the upper atmosphere in the presence of higher energy UV light (70). Due to this specific formation pathway, Δ^200^Hg is linked to long-range transport, conservative upon deposition, susceptible only to dilution, and thus has become a relative tracer of the effect of far-field atmospheric Hg to an ecosystem (13, 18, 23, 70). These lake trout Δ^200^Hg values were remarkably constant, albeit elevated, (0.09 ± 0.02‰) (*SI Appendix*, Fig. S5) throughout the study. In addition, Δ^200^Hg values for both the Manitou core (0.06 ± 0.01‰) and southern basin core (0.04 ± 0.01‰) were constant through time.

The lack of annual variation is perplexing considering the profound changes in United States and global Hg mitigation strategies. We recognize though, that we do not fully understand drivers in variability of Δ^200^Hg. We can only conclude that the Hg sources mitigated regionally had little effect on Δ^200^Hg values in sediment and lake trout; therefore, the source of Δ^200^Hg to fish did not change in the Great Lakes region over the study period. To more completely understand these results, more work on Δ^200^Hg formation and transport is necessary.

### Recalibrating Our Interpretation of Archives.

Previous work has shown that fish and sediment archives agree well with emission inventories when studies investigate persistent organic pollutants, such as polychlorinated biphenyls, dieldrin, and chlordane (71). Except for the period from 1972 to 1988, we found that Hg concentrations in lake trout do not agree well with declined emission inventories or Hg deposition to the sediment. Unlike many organic contaminants, MeHg has historically been naturally present in the environment, and anthropogenic activity has exacerbated the amount of actively cycling Hg. Our study shows that source reductions of Hg have altered the Hg isotopic composition of predatory fish, but food web shifts have at least temporarily offset a beneficial effect on fish bioaccumulation. We could not have made this conclusion without analyzing Hg, C, and N stable isotopes. During Hg source reductions, lake trout energetic pathways shifted either directly or indirectly to the benthos, which has dampened the expected reduction in MeHg concentrations in fish (28). This is additionally due to lake trout growth rate decreases (52). Furthermore, we have shown that sediment cores, often applied to show success of Hg mitigation strategies, do not predict MeHg trends in fish because they are inadequate to trace complex food web perturbations and the effect of Hg mitigation on Hg bioaccumulated in fish. These results demonstrate the importance of the route of delivery for bioavailable Hg, as reductions to emissions impacted Hg isotopic shifts in fish 3 to 4 times greater than sediments.

The rapid rate of δ^202^Hg response in Lake Michigan from 1988 to 1992 (Fig. 2*B*) indicates a near-field Hg source shift to the Hg deposited to the lake, and the synchronous shift between separate basins (Fig. 2*C*) indicates a broadly distributed Hg regional source. The large increase in δ^202^Hg (+0.6‰; ρ = 0.81) beginning in 1988 provides evidence that reductions to domestic emissions affects fish more rapidly than previously recognized; however, it is perplexing that HgT concentration did not immediately decrease in parallel. In the North American atmosphere, researchers are observing faster than expected reductions in domestic atmospheric Hg inventories (11) resulting from reduced United States emissions. In Lake Michigan, fish δ^202^Hg responded more rapidly than expected due to the late 1980s shift in source inputs of Hg and to a greater degree than sediments.

Our research reveals that it is possible to detect source-specific Hg reductions in fish archives by incorporating isotopic analyses. This would not have been possible by assessing HgT concentration only. Independent of Hg emissions, Hg concentrations in fish responded to an ecosystem perturbation resulting from invasive dreissenid mussels that without the aid of the combined Hg, C, and N stable isotopes ratios, would provide a false impression that reduced Hg emissions are no longer benefiting Hg concentrations in fish. From the literature, we know that during the mid to late 1990s, prey quality decreased (46, 50) due to decreasing *Diporeia* populations (38), and temporarily lake trout lipid content decreased (*SI Appendix*, Fig. S2), which led to increasing HgT concentrations in fish but stable C, N, and Hg isotope ratios. This, along with the invasion of round gobies, led to a dietary shift by lake trout toward round gobies and resulted in changes to stable C, N, and Hg isotope ratios. Furthermore, because the ratios of δ^202^Hg and Δ^199^Hg responded in opposite directions, we can conclude that lake trout in 2012 are bioaccumulating a different Hg source portfolio in Lake Michigan relative to the 1970s. Hg concentrations in fish following energetic shifts in the food web would be higher if not for reduction in Hg emissions at the domestic level. For decision-makers and natural resource managers, it is crucial to be aware that Hg source control and MeHg bioaccumulation are not intrinsically linked, as demonstrated by the lack of monotonic decline in Hg concentrations in fish from this study period. Many other factors [e.g., dietary shifts, water quality, biogenetics, and fish age (4)] can affect fish Hg concentration beyond input rates, highlighting the value of this fish archive and the necessity of additional isotopic information to interpret source reductions. While specific source inputs may be declining due to Hg mitigation, declines in HgT concentrations in lake trout may be counteracted by shifts in dietary sources of Hg.

## Materials and Methods

### Sample Collection.

Field sampling protocols for the GLFMSP have been documented elsewhere (72, 73). Generally, sites were visited on a biennial basis, where 1 site was intended to represent a shallow, southern location (Saugatuck, Michigan; 61 m) and another a deep, northern location (Sturgeon Bay, Wisconsin; 119 m) (*SI Appendix*, Fig. S3). In early years of the survey, a site in near Charlevoix, Michigan (61 m) was also sampled. Since 1978, 5 similarly sized whole lake trout (600 to 700 mm) were composited to create 1 sample and 10 unique samples were created at each collection location. From this collection of 10 composites, 1 to 5 composite samples were randomly chosen per year based on availability. In instances where only 1 composite was available, the data were reported as points rather than a boxplot.

Archived lake trout samples obtained from the GLFMSP were removed from freezers (−20 °C) and allowed to thaw until a Teflon-coated stainless-steel spatula could mix and then remove enough partially frozen mass sufficient for the necessary analyses. Subsamples were lyophilized at the US Geological Survey Mercury Research Laboratory (USGS MRL) in Middleton, WI. Unlike previous studies investigating HgT in Great Lakes trout (23, 42, 51), we chose to analyze on a dry weight basis to avoid the complexities associated with water content in older (1970s) samples.

In 2009 and 2010, sediment cores were collected using clean metal techniques in the southern basin and northern Manitou pass (*SI Appendix*, Fig. S2). Cores were collected from the US EPA R/V *Lake Guardian* with a box corer, then subcored and sectioned onboard and frozen. Frozen samples were then lyophilized. Age dating was performed at the St. Croix Watershed Research Station, Science Museum of Minnesota, using ^210^Pb decay (measured via ^210^Po) and a constant rate of supply model was applied to estimate sediment age and dry mass accumulation using previously established methods (2, 74).

### Sample (HgT) Determination and Hg Isotope Preparation.

Fish composite samples were weighed into borosilicate vials and digested with concentrated nitric acid (5 mL) for 8 h at 90 °C. Then samples were cooled and concentrated BrCl (10% [vol/vol]) was added to completely oxidize MeHg into inorganic Hg. Samples were then heated for an additional 8 h (90 °C). The resulting solutions were diluted to a 50% acid concentration and quantified for HgT using an adaptation of US EPA method 1631 (18, 20, 75). Briefly, hydroxylamine was used to reduce the oxidative capacity of BrCl, followed by stannous reduction, gold amalgamation, and thermal release. Atomic florescence was used to quantify HgT. A thorough dataset ensuring the digest precision and accuracy may be found in *SI Appendix*, Table S3. Secondary standard recoveries were 101 ± 4% and spiked recoveries, 100 ± 5% (max 106% and min 92%). To determine whether inorganic Hg was a substantial proportion of the HgT, 30 randomly chosen fish, spanning the entirety of this dataset, were digested for MeHg (76). In all instances MeHg content was within 5% of the HgT concentration and quality control and assurance met or exceeded the USGS MRL criteria (https://wi.water.usgs.gov/mercury-lab).

Sediments were similarly processed by weighing samples into borosilicate vials and digesting with aqua regia (5 mL) overnight at 90 °C. Following digestion, cooled solutions were diluted to 50% acid and quantified in a manner consistent with the previously mentioned fish. To ensure precision and accuracy, 5% of the sample count was represented by standard reference material International Atomic Energy Agency (IAEA) SL1 (130 ng g^−1^). Recoveries of standard reference materials were 100% (1 SD = 0.02%); triplicate relative SDs were 3 to 4%.

### Hg Stable Isotope Ratios.

Using the appropriate aliquot to make an approximate 1.5 ng Hg mL^−1^ solution, samples were pipetted into polypropylene vials. When necessary, matrix matching acid was added to ensure a consistent matrix was shared among samples. The sample introduction process, laboratory protocol, and instrument setup have been thoroughly described previously (77). Briefly, using a matrix-matched NIST 3133, samples were measured for Hg isotopes following the standard sample bracketing, Tl (NIST 997) was used for mass bias correction, and Hg gas was produced by stannous reduction over a custom designed gas–liquid separator (18, 77). We followed previous convention (78) by expressing MDF in terms of δ^xxx^Hg and mass independent fractionation of Hg in odd isotopes and even isotopes as Δ^199^Hg and Δ^200^Hg, respectively (78). Samples were consistently within 10% (averaged 0 ± 6%) of the NIST concentration, and UM-Almaden was measured in 20% of samples to ensure instrument stability and accuracy. Isotopic results of UM-Almaden (δ^202^Hg: −0.51± 0.04‰, Δ^199^Hg: 0.00 ± 0.03‰, Δ^200^Hg: −0.01 ± 0.01‰, and Δ^204^Hg:0.01 ± 0.03 to 1 SD) and IAEA SL1 (δ^202^Hg: −1.27 ± 0.03‰, Δ^199^Hg: −0.17 ± 0.05‰, Δ^200^Hg: 0.01 ± 0.03‰, and Δ^204^Hg: −0.04 ± 0.05‰ to 1 SD) were consistent with previous findings (18, 77). Triplicate results are in *SI Appendix*, Table S3 and the replication of sample triplicates indicated that the Δ^204^Hg patterns observed are substantial and not simply the result of analytical variance of each Hg isotope ratio.

### C, N Stable Isotope Ratios.

For C and N stable isotope analysis, 1.00 ± 0.10 mg (dry mass) of each composite fish tissue sample was weighed into a tin capsule; samples were analyzed at the University of California, Davis Stable Isotope Facility. Results are reported as δ values (δ^13^C, δ^15^N) using Vienna Peedee Belemnite as the standard for δ^13^C and atmospheric nitrogen as the standard for δ^15^N. Laboratory standards included G-13 (bovine liver), G-18 (nylon 5), G-20, (glutamic acid), and G-21 (enriched alanine); mean stable isotope values matched reference values within 0.03‰ and precision was <0.08‰ (1 SD). Triplicates were added to determine precision (results in *SI Appendix*, Table S4). The resulting error from replication was not substantial compared to the changes in C and N isotope ratios, confirming that the observations here are not the result of analytical variance. Because sample differences in lipid content can bias δ^13^C values as a result of differences in fractionation between lipids and protein, sample δ^13^C values should be normalized for lipid content (79). We corrected δ^13^C values for variable lipid content using an arithmetic, mass-balance correction (79) (*SI Appendix*, Eq. **S1**).

## Supplementary Material

Supplementary File
