# Supplementary material for: Mercury source changes and food web shifts alter contamination signatures of predatory fish from Lake Michigan
Source: Proc Natl Acad Sci U S A. 2019 Nov 4;116(47):23600–8. doi: 10.1073/pnas.1907484116 (PMC6876223; doi:10.1073/pnas.1907484116)
Supplement: Supplementary File [file pnas.1907484116.sapp.pdf]

PNAS
www.pnas.org

Supplementary Information for

Mercury Source Changes and Food Web Shifts Alter Contamination Signatures of Predatory Fish from Lake Michigan

Ryan F. Lepak, Joel C. Hoffman, Sarah E. Janssen, David P. Krabbenhoft, Jacob M. Ogorek, John F. DeWild, Michael T. Tate, Christopher L. Babiarz, Runsheng Yin, Elizabeth W. Murphy, Daniel R. Engstrom and James P. Hurley

James P. Hurley

Email: jphurley@wisc.edu

This PDF file includes:

- Supplementary text
- Equation S1
- Figs. S1 to S5
- Tables S1 to S5
- References for SI reference citations

Other supplementary materials for this manuscript include the following:

None

Supplementary Information Text

Lipid correction model for $\delta^{13}C$. Lipid correction of $\delta^{13}C$ is essential in fish with widely varying lipid content because the formation of lipids results in a negative $\delta^{13}C$ fractionation which can affect the bulk signature when comparing between individual fish and species. To better ascertain the carbon energy pathways of the fish, various correctional methods are used based on either analytical or mathematical methods[1,2]. Here, the latter is used and in the absence of lipid content, $C:N_{bulk}$ ratio is used as a proxy as suggested by Fry et al[3].

$$\boldsymbol{\delta^{13}C_{lipid\ free} = \delta^{13}C_{bulk} + (\Delta\delta^{13}C_{lipid} * \left(C:N_{lipid\ free} - C:N_{bulk}\right))/C:N_{bulk}} \quad \text{(Equation S1)}$$

Where the $\Delta\delta^{13}C_{lipid}$ (-6.5 ‰) is the recommended constant for fish muscles with a $C:N_{bulk}$ ranging 3.4 to 15.3 and $C:N_{lipid}$ (3.4) is the molar ratio of free lipids. From this, percent lipids can be estimated using the molar C:N ratio[1,2].

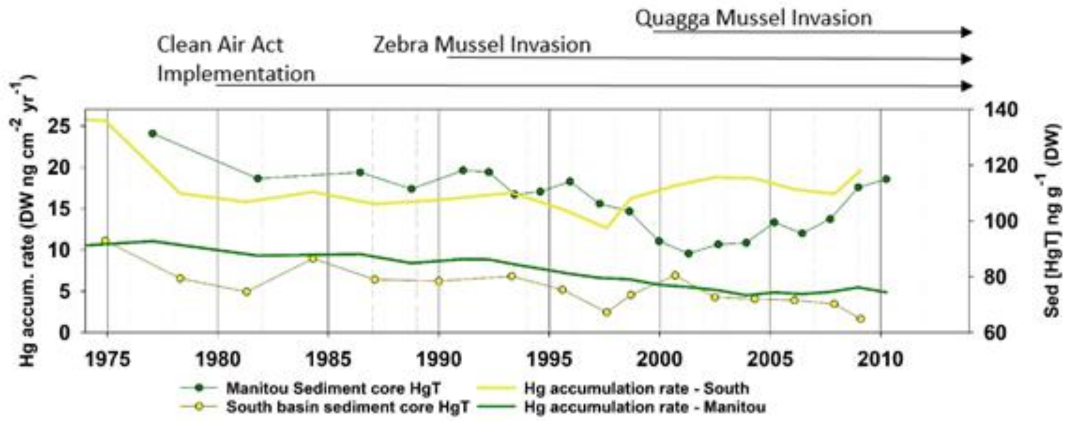

Fig. S1. Total mercury (HgT ng g^{-1} dry weight [DW]) and mercury (Hg) accumulation rate (ng cm^{-2} yr^{-1}) for two cores collected in Lake Michigan.

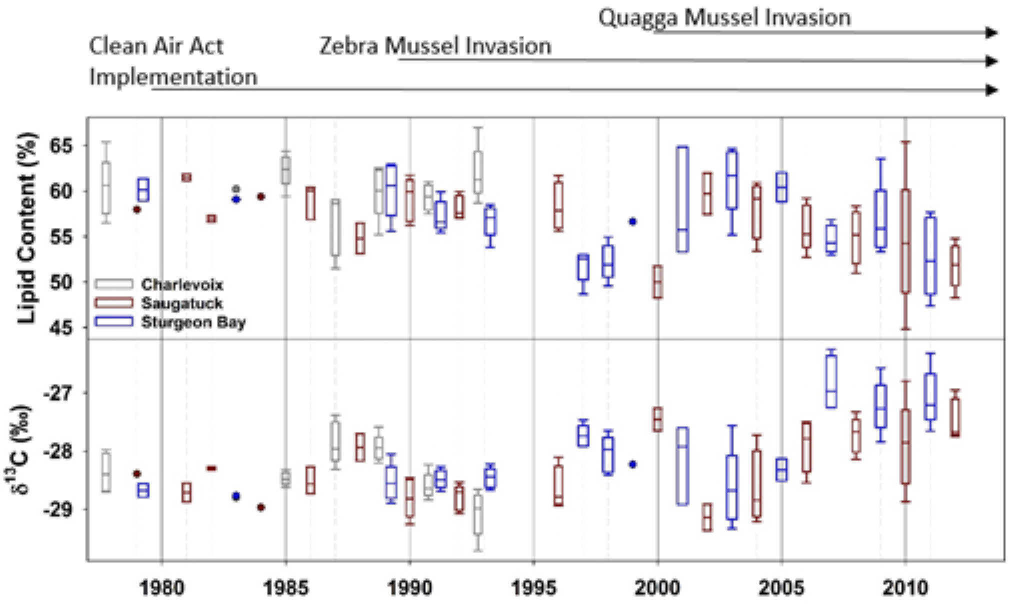

Figure S2. (Top): Percent lipid content in lake trout calculated by the methods mentioned in supplemental text and elsewhere[1,2]. (Bottom): The measured $\delta^{13}C$ in lake trout. Each data point represents a composite of 5 lake trout, and the boxplots were used when 3-5 unique composites were measured within a single year. Boxplots indicate the mean and quartiles of the fish composites sampled in a site and year. Whiskers represent the 10th and 90th percentiles. indicate the mean and quartiles of the fish composites sampled in a site and year. Plot color indicates site with gray, red and blue representing Charlevoix, Saugatuck and Sturgeon Bay respectively.

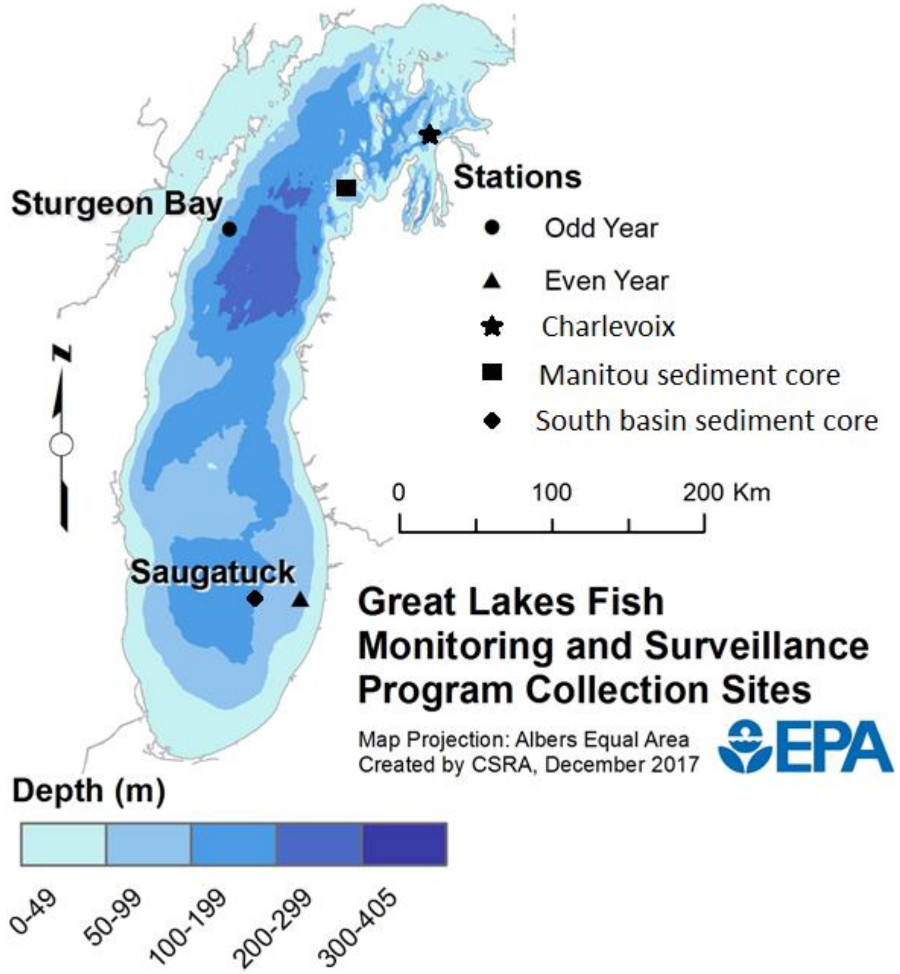

Fig. S3. Sampling map of the Great Lakes Fish Monitoring and Surveillance Program (GLFMSP) collection sites along with sediment coring sites and bathymetry.

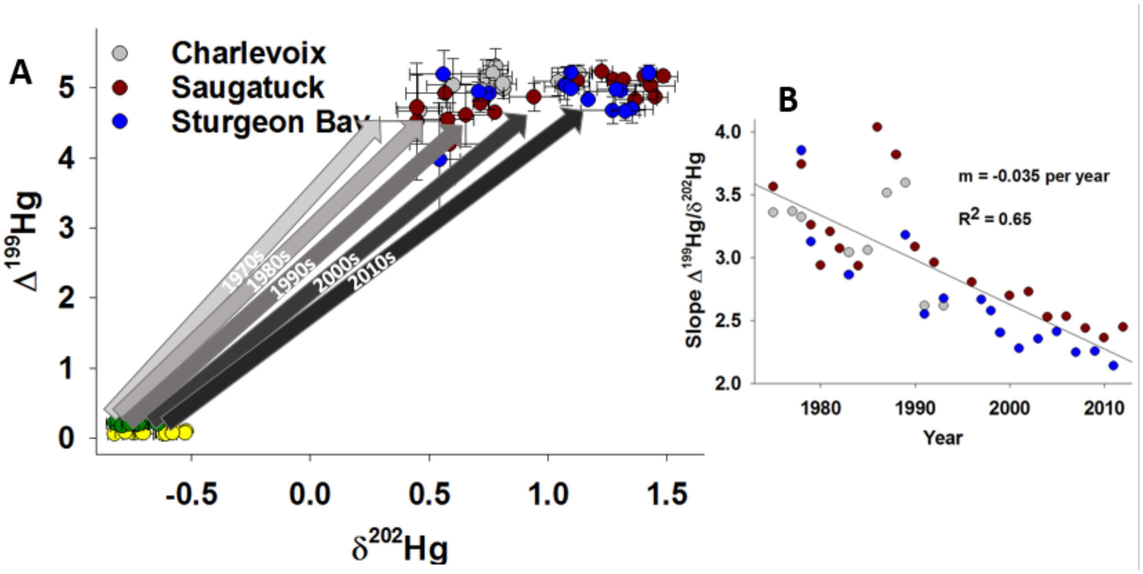

Figure S4. A temporal comparison of the sediments and fish represented in Figure 2. Here, the slope of $\Delta^{199}Hg:\delta^{202}Hg$ (traditionally = 2.4) is used to predict whether sediment Hg might contribute to fish Hg. This slope is resultant of the fractionation in Hg isotopes due to photochemical demethylation[4-7]. When the slope is 2.4, the prediction can be made that the source of Hg in fish is linked to sediments (typically as sedimentary flux of MeHg). Here, only in the recent 2010s does this slope reflect 2.4 but we hypothesize this is not because sedimentary fluxes of MeHg affect fish Hg isotope ratios but rather that remnant sedimentary Hg now most closely resembles Hg bioaccumulated in fish from the environment. Panels A and B emphasize that prior to 2010s lake sediments were poor predictors of fish Hg sources.

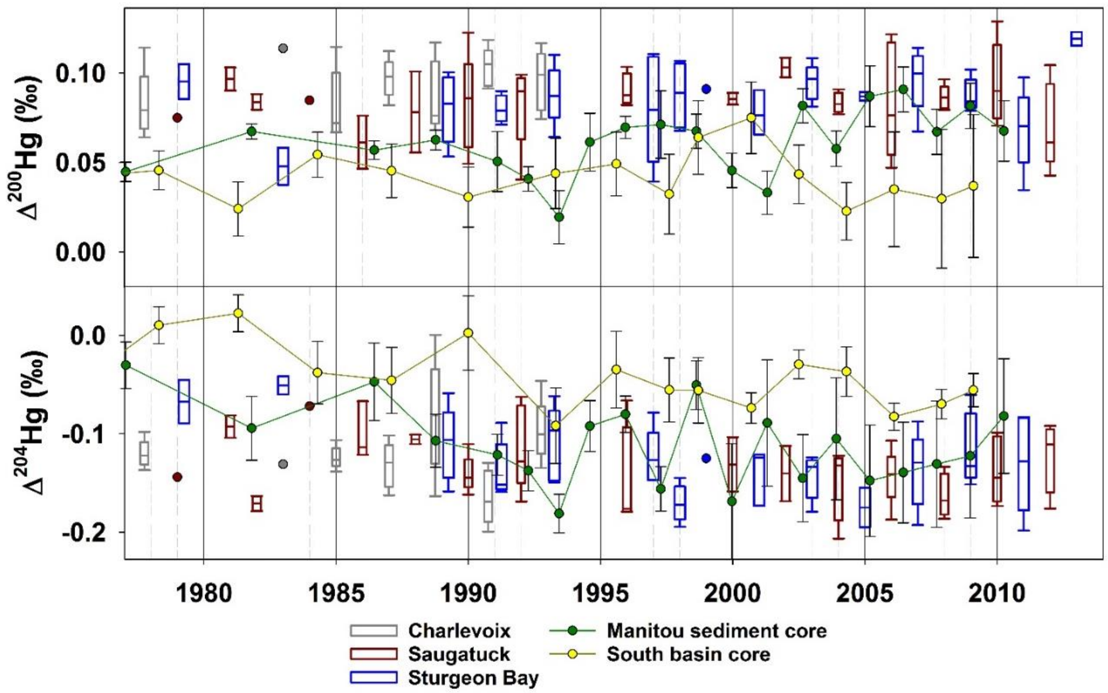

Figure S5. The even-mass independent fractionation values for lake trout and 2 sediment cores. Each data point represents a composite of 5 lake trout and the boxplots were used when 3-5 unique composites were measured within a single year. Box plots indicate the mean and quartiles of the fish composites sampled in a site and year. Plot color indicates site with gray, red, and blue representing Charlevoix, Saugatuck and Sturgeon Bay respectively.

Table S1. Hg, C and N stable isotope ratios for the lake trout composites along with the d^{13}C lipid free, C:N$_{bulk}$ and length and weight measurements (next 3 pages) Site names LM-C, LM-St and LM-Sa represent Charlevoix, Sturgeon Bay and Saugatuck, respectively. USGS ID is the unique US Geological Survey identifier code.

Site	USGS ID	Year	[HgT] ng g^{-1}	δ^{202}Hg ‰	Δ^{199}Hg ‰	Δ^{200}Hg ‰	Δ^{201}Hg ‰	Δ^{204}Hg ‰	δ^{13}C ‰	$\delta^{13}C_{lipid-free}$ ‰	δ^{15}N ‰	Molar C:N
LM-C	MSC712AA	1978	435.2	0.70	5.36	0.11	4.18	-0.13	-28.67	-24.46	14.50	10.8
LM-Sa	MSC727AA	1979	307.8	0.60	4.40	0.06	3.47	-0.11	-28.41	-24.51	14.03	9.5
LM-Sa	MSC728AA	1979	419.4	0.52	4.32	0.08	3.34	-0.12	-28.29	-24.49	14.05	9.1
LM-Sa	MSC731AA	1979	355.9	0.61	4.17	0.06	3.27	-0.06	-28.70	-24.53	14.20	10.6
LM-St	MSC736AA	1979	370.6	0.64	4.53	0.08	3.49	-0.12	-28.11	-24.38	14.25	8.9
LM-Sa	MSC744AA	1981	423.1	0.66	4.71	0.11	3.67	-0.17	-28.22	-24.34	13.54	9.5
LM-C	MSC749AA	1983	244.2	0.81	4.98	0.11	3.81	-0.13	-28.80	-24.63	13.59	10.6
LM-St	MSC750AA-ave	1983	392.3	0.61	4.54	0.06	3.48	-0.06	-28.77	-24.68	13.77	10.2
LM-Sa	MSC752AA	1984	388.3	0.78	4.65	0.08	3.57	-0.07	-28.97	-24.86	14.18	10.3
LM-C	MSC753AA	1985	266.1	0.84	4.97	0.11	3.81	-0.13	-28.42	-24.31	13.96	10.3
LM-C	MSC754AA	1985	247.3	0.73	4.78	0.07	3.65	-0.12	-28.52	-24.12	14.22	11.7
LM-C	MSC755AA	1985	292.1	0.77	5.18	0.07	3.99	-0.14	-28.32	-23.99	13.97	11.4
LM-C	MSC756AA	1985	230.3	0.85	5.01	0.07	3.88	-0.13	-28.49	-24.15	14.13	11.4
LM-C	MSC757AA	1985	267.7	0.86	5.32	0.09	4.05	-0.11	-28.62	-24.13	13.56	12.3
LM-Sa	MSC758AA	1986	462.6	0.51	5.06	0.08	3.95	-0.07	-28.73	-24.54	13.81	10.7
LM-Sa	MSC759AA	1986	509.3	0.42	4.71	0.05	3.69	-0.11	-28.27	-24.36	13.78	9.5
LM-Sa	MSC760AA-ave	1986	573.1	0.54	5.04	0.06	3.96	-0.12	-28.57	-24.41	14.09	10.6
LM-C	MSC762AA	1987	498.3	0.69	5.32	0.11	4.12	-0.13	-27.62	-23.91	13.96	8.9
LM-C	MSC763AA	1987	300.5	0.45	4.45	0.09	3.49	-0.10	-28.31	-24.23	14.13	10.2
LM-C	MSC764AA	1987	407.3	0.68	5.23	0.10	4.06	-0.12	-28.02	-23.97	14.18	10.1
LM-C	MSC765AA	1987	396.1	0.68	5.32	0.10	4.12	-0.16	-27.96	-23.89	14.31	10.2
LM-C	MSC766AA	1987	282.5	0.52	4.86	0.08	3.80	-0.15	-27.38	-23.90	14.04	8.2
LM-Sa	MSC767AA	1988	554.5	0.69	5.06	0.10	3.93	-0.10	-27.70	-24.08	13.94	8.6
LM-Sa	MSC768AA	1988	536.3	0.45	4.78	0.06	3.78	-0.11	-28.17	-24.29	14.35	9.4
LM-C	MSC769AA	1989	248.8	0.41	4.78	0.08	3.77	-0.10	-27.94	-23.62	14.16	11.3
LM-C	MSC770AA-ave	1989	233.2	0.42	4.67	0.10	3.67	-0.08	-27.94	-23.80	14.29	10.5
LM-C	MSC771AA	1989	305.0	0.42	4.65	0.12	3.63	-0.16	-28.07	-23.91	14.62	10.5
LM-C	MSC772AA	1989	273.6	0.41	4.66	0.07	3.66	-0.07	-28.20	-23.85	14.35	11.5
LM-C	MSC773AA	1989	244.8	0.63	4.58	0.08	3.61	0.00	-27.59	-23.81	13.91	9.1
LM-St	MSC774AA	1989	322.9	0.71	4.95	0.09	3.86	-0.06	-28.55	-24.47	14.16	10.2
LM-St	MSC775AA	1989	349.2	0.74	4.89	0.07	3.84	-0.10	-28.89	-24.54	14.21	11.5
LM-St	MSC776AA	1989	366.3	0.78	5.11	0.10	3.98	-0.16	-28.48	-24.28	14.34	10.7
LM-St	MSC777AA	1989	313.9	0.65	4.75	0.08	3.69	-0.11	-28.05	-24.24	14.40	9.2
LM-St	MSC778AA	1989	384.5	0.66	5.04	0.05	4.00	-0.13	-28.74	-24.35	14.26	11.7
LM-Sa	MSC779AA	1990	369.2	0.87	5.05	0.05	3.95	-0.11	-28.46	-24.53	14.10	9.6
LM-Sa	MSC780AA-ave	1990	318.8	0.91	4.86	0.09	3.81	-0.14	-28.82	-24.60	14.03	10.8
LM-Sa	MSC781AA	1990	231.9	1.12	4.96	0.07	3.87	-0.14	-29.26	-24.97	14.28	11.2
LM-Sa	MSC782AA	1990	310.2	0.86	4.54	0.09	3.54	-0.16	-28.99	-24.84	14.60	10.5
LM-Sa	MSC783AA	1990	359.7	0.96	4.93	0.12	3.85	-0.15	-28.51	-24.65	13.96	9.4
LM-C	MSC784AA	1991	194.2	1.14	5.21	0.11	4.10	-0.15	-28.64	-24.40	14.44	10.9
LM-C	MSC785AA	1991	183.5	1.10	5.23	0.12	4.12	-0.20	-28.58	-24.47	14.10	10.3
LM-C	MSC786AA	1991	216.9	1.03	5.25	0.10	4.16	-0.17	-28.23	-24.27	13.97	9.7
LM-C	MSC787AA	1991	203.8	0.87	4.80	0.09	3.78	-0.13	-28.69	-24.66	14.30	10.0
LM-C	MSC788AA	1991	191.3	1.07	4.95	0.09	3.87	-0.18	-28.84	-24.66	14.56	10.7

Table S1. continued

Site	USGS ID	Year	[HgT]	δ^{202}Hg	Δ^{199}Hg	Δ^{200}Hg	Δ^{201}Hg	Δ^{204}Hg	δ^{13}C	$\delta^{13}C_{lipid\text{-}free}$	δ^{15}N	Molar C:N
			ng g^{-1}	‰	‰	‰	‰	‰	‰	‰	‰	
LM-St	MSC789AA	1991	297.9	1.09	5.14	0.09	4.03	-0.15	-28.27	-24.47	14.22	9.1
LM-St	MSC790AA-ave	1991	250.7	1.07	5.17	0.09	4.04	-0.15	-28.55	-24.66	14.34	9.5
LM-St	MSC791AA	1991	287.2	1.09	4.97	0.07	3.89	-0.16	-28.69	-24.54	14.63	10.5
LM-St	MSC792AA	1991	215.9	1.00	4.71	0.08	3.71	-0.09	-28.49	-24.51	14.07	9.8
LM-St	MSC793AA	1991	296.2	1.09	5.15	0.08	4.02	-0.13	-28.39	-24.52	14.14	9.4
LM-Sa	MSC794AA	1992	321.6	1.13	5.21	0.09	4.08	-0.13	-28.70	-24.55	14.25	10.5
LM-Sa	MSC795AA	1992	369.0	1.09	5.20	0.09	4.06	-0.08	-28.69	-24.61	14.33	10.2
LM-Sa	MSC796AA	1992	307.8	1.16	4.99	0.10	3.88	-0.06	-28.96	-25.00	14.21	9.8
LM-Sa	MSC797AA	1992	285.0	1.18	4.95	0.10	3.86	-0.17	-29.07	-25.14	14.46	9.6
LM-Sa	MSC798AA	1992	245.3	1.06	5.15	0.04	3.97	-0.13	-28.54	-24.61	14.03	9.6
LM-C	MSC799AA	1993	258.9	1.08	5.07	0.12	4.00	-0.05	-29.14	-24.89	14.80	11.0
LM-C	MSC800AA-ave	1993	287.8	1.08	5.08	0.08	3.97	-0.10	-28.66	-24.60	14.81	10.1
LM-C	MSC801AA	1993	233.9	1.22	5.25	0.10	4.13	-0.13	-28.86	-24.63	14.56	10.9
LM-C	MSC802AA	1993	182.2	1.13	5.38	0.07	4.16	-0.11	-28.98	-24.70	14.53	11.2
LM-C	MSC803AA	1993	238.1	1.15	5.23	0.10	4.11	-0.10	-29.71	-25.02	14.49	13.6
LM-St	MSC804AA	1993	335.7	0.98	5.31	0.06	4.06	-0.10	-28.66	-24.63	14.62	10.0
LM-St	MSC805AA	1993	218.8	1.08	5.20	0.11	4.03	-0.06	-28.38	-24.49	14.72	9.5
LM-St	MSC806AA	1993	297.7	1.20	5.14	0.09	4.02	-0.09	-28.23	-24.56	14.15	8.7
LM-St	MSC807AA	1993	317.3	1.10	5.17	0.09	4.07	-0.15	-28.44	-24.51	14.52	9.6
LM-St	MSC808AA	1993	293.3	1.11	5.28	0.09	4.12	-0.15	-28.58	-24.60	14.51	9.8
LM-Sa	MSC810AA-ave	1996	315.5	1.27	5.41	0.10	4.26	-0.18	-28.85	-24.56	14.89	11.2
LM-Sa	MSC811AA	1996	313.9	1.23	5.32	0.09	4.10	-0.17	-28.11	-24.30	14.33	9.2
LM-Sa	MSC812AA	1996	298.5	1.17	5.09	0.08	3.96	-0.18	-28.94	-24.87	14.61	10.1
LM-Sa	MSC813AA	1996	368.0	1.23	5.11	0.09	4.04	-0.07	-28.72	-24.80	14.54	9.6
LM-St	MSC814AA	1997	324.5	1.19	5.05	0.11	3.97	-0.15	-27.74	-24.14	13.89	8.5
LM-St	MSC815AA	1997	416.9	1.12	4.91	0.06	3.85	-0.08	-27.91	-24.30	14.81	8.5
LM-St	MSC816AA	1997	315.2	1.08	4.95	0.08	3.90	-0.15	-27.47	-24.21	14.80	7.6
LM-St	MSC817AA	1997	353.4	1.09	4.73	0.04	3.68	-0.13	-27.65	-24.14	14.48	8.3
LM-St	MSC818AA	1997	318.4	0.92	5.47	0.11	4.25	-0.12	-27.92	-24.35	14.46	8.4
LM-St	MSC819AA	1998	631.7	1.12	5.05	0.09	3.95	-0.14	-27.65	-24.32	14.46	7.8
LM-St	MSC820AA-ave	1998	456.2	1.14	5.04	0.10	3.96	-0.17	-28.41	-24.65	14.62	9.0
LM-St	MSC821AA	1998	578.4	1.08	4.94	0.11	3.85	-0.16	-28.31	-24.71	14.84	8.5
LM-St	MSC822AA	1998	450.0	1.09	4.91	0.07	3.79	-0.18	-27.97	-24.46	14.30	8.3
LM-St	MSC823AA	1998	583.9	1.07	5.00	0.07	3.88	-0.19	-27.88	-24.39	14.20	8.2
LM-St	MSC824AA	1999	438.8	1.17	4.83	0.09	3.73	-0.13	-28.23	-24.34	15.13	9.5
LM-Sa	MSC825AA	2000	508.3	1.26	5.10	0.08	4.00	-0.16	-27.65	-24.15	14.48	8.2
LM-Sa	MSC826AA	2000	616.0	1.29	5.13	0.09	3.96	-0.10	-27.26	-24.04	14.51	7.5
LM-St	MSC827AA	2001	653.7	1.20	4.95	0.07	3.81	-0.17	-27.92	-24.10	14.80	9.2
LM-St	MSC828AA	2001	645.1	1.24	5.01	0.08	3.90	-0.12	-27.60	-23.97	14.77	8.6
LM-St	MSC830AA-ave	2001	389.0	1.48	4.89	0.09	3.80	-0.12	-28.92	-24.39	14.69	12.5
LM-Sa	MSC831AA	2002	693.5	1.37	5.11	0.11	4.01	-0.11	-29.36	-25.05	14.32	11.3
LM-Sa	MSC832AA	2002	812.3	1.27	5.11	0.10	4.00	-0.17	-28.91	-24.95	14.68	9.7
LM-St	MSC833AA	2003	376.5	1.46	5.19	0.10	4.08	-0.15	-29.01	-24.56	14.31	12.1
LM-St	MSC834AA	2003	438.3	1.44	5.29	0.11	4.14	-0.13	-28.60	-24.31	14.68	11.2

Table S1. continued

Site	USGS ID	Year	[HgT] ng g^{-1}	δ^{202}Hg ‰	Δ^{199}Hg ‰	Δ^{200}Hg ‰	Δ^{201}Hg ‰	Δ^{204}Hg ‰	δ^{13}C ‰	$\delta^{13}C_{lipid\text{-}free}$ ‰	δ^{15}N ‰	Molar C:N
LM-St	MSC835AA	2003	345.5	1.41	5.01	0.08	3.92	-0.13	-28.67	-24.44	14.38	10.9
LM-St	MSC836AA	2003	446.0	1.44	5.28	0.10	4.12	-0.18	-27.56	-23.78	14.36	9.1
LM-St	MSC837AA	2003	304.0	1.38	5.27	0.09	4.12	-0.12	-29.33	-24.82	13.77	12.4
LM-Sa	MSC838AA	2004	296.0	1.44	5.30	0.09	4.08	-0.21	-27.73	-24.09	13.61	8.6
LM-Sa	MSC839AA	2004	421.2	1.38	5.07	0.08	3.94	-0.13	-28.87	-24.80	13.73	10.2
LM-Sa	MSC841AA	2004	441.3	1.41	5.23	0.08	4.06	-0.12	-29.21	-24.98	14.19	10.9
LM-Sa	MSC842AA	2004	522.5	1.39	5.07	0.08	3.92	-0.13	-28.81	-24.70	14.67	10.3
LM-St	MSC843AA	2005	444.8	1.23	4.94	0.09	3.84	-0.16	-28.13	-24.07	14.17	10.1
LM-St	MSC844AA	2005	411.4	1.34	5.00	0.08	3.83	-0.19	-28.51	-24.20	13.74	11.3
LM-Sa	MSC845AA	2006	208.8	1.29	4.81	0.05	3.74	-0.14	-27.50	-23.91	13.09	8.5
LM-Sa	MSC846AA	2006	342.5	1.39	5.08	0.06	3.90	-0.19	-27.78	-24.00	13.67	9.1
LM-Sa	MSC847AA	2006	432.7	1.55	5.09	0.11	4.05	-0.14	-28.54	-24.56	14.11	9.8
LM-Sa	MSC848AA	2006	397.4	1.48	5.08	0.12	4.06	-0.11	-27.55	-23.80	13.18	9.0
LM-Sa	MSC849AA	2006	334.1	1.46	5.08	0.08	3.99	-0.14	-28.17	-24.08	13.55	10.3
LM-St	MSC850AA-ave	2007	339.6	1.37	4.91	0.07	3.82	-0.19	-27.25	-23.44	14.34	9.2
LM-St	MSC851AA	2007	498.9	1.35	4.86	0.11	3.80	-0.12	-27.24	-23.53	14.86	8.9
LM-St	MSC852AA	2007	486.9	1.06	4.61	0.10	3.61	-0.09	-26.97	-23.06	14.19	9.5
LM-St	MSC853AA	2007	472.6	1.21	4.48	0.10	3.52	-0.15	-26.25	-22.65	14.12	8.5
LM-St	MSC854AA	2007	342.8	1.38	4.54	0.10	3.54	-0.13	-26.48	-22.81	15.04	8.7
LM-Sa	MSC855AA	2008	382.0	1.47	5.23	0.09	4.07	-0.13	-27.33	-23.88	13.60	8.1
LM-Sa	MSC856AA	2008	324.3	1.49	5.10	0.09	3.99	-0.19	-27.67	-24.06	13.32	8.5
LM-Sa	MSC857AA	2008	398.8	1.58	5.23	0.08	4.11	-0.17	-28.14	-24.11	13.02	10.0
LM-Sa	MSC858AA	2008	450.9	1.41	5.18	0.10	4.02	-0.18	-27.90	-23.97	12.92	9.6
LM-Sa	MSC859AA	2008	276.6	1.47	5.07	0.08	3.95	-0.15	-27.59	-23.81	13.15	9.1
LM-St	MSC860AA-ave	2009	385.3	1.44	4.89	0.08	3.82	-0.15	-27.84	-23.41	15.10	11.9
LM-St	MSC861AA	2009	324.4	1.39	4.75	0.09	3.66	-0.10	-27.16	-23.45	14.60	8.9
LM-St	MSC862AA	2009	342.0	1.22	4.37	0.08	3.36	-0.06	-26.58	-22.94	14.57	8.6
LM-St	MSC863AA	2009	465.9	1.33	4.79	0.10	3.74	-0.13	-27.35	-23.46	14.42	9.5
LM-St	MSC864AA	2009	330.7	1.40	4.70	0.08	3.66	-0.14	-27.27	-23.44	13.98	9.3
LM-Sa	MSC865AA	2010	312.0	1.39	4.84	0.08	3.77	-0.14	-27.86	-24.10	13.07	9.0
LM-Sa	MSC866AA	2010	337.8	1.44	4.88	0.10	3.80	-0.17	-27.79	-24.19	13.10	8.5
LM-Sa	MSC867AA	2010	248.8	1.44	4.90	0.13	3.79	-0.11	-26.80	-23.86	13.11	6.9
LM-Sa	MSC868AA	2010	436.7	1.45	4.89	0.07	3.80	-0.16	-28.87	-24.30	13.44	12.8
LM-Sa	MSC869AA	2010	510.4	1.53	4.82	0.09	3.76	-0.10	-28.25	-24.54	14.36	8.8
LM-St	MSC870AA-ave	2011	273.7	1.40	4.80	0.10	3.71	-0.13	-27.21	-23.66	13.90	8.4
LM-St	MSC871AA	2011	258.9	1.27	4.50	0.07	3.53	-0.08	-27.26	-23.29	13.62	9.8
LM-St	MSC872AA	2011	211.7	1.36	4.67	0.07	3.63	-0.08	-26.32	-23.17	14.33	7.4
LM-St	MSC873AA	2011	226.6	1.27	4.59	0.08	3.59	-0.20	-27.03	-23.67	13.38	7.9
LM-St	MSC874AA	2011	258.3	1.32	4.74	0.03	3.69	-0.16	-27.66	-23.77	13.99	9.4
LM-Sa	MSC875AA	2012	359.8	1.40	4.99	0.08	3.87	-0.14	-27.68	-23.94	12.75	9.0
LM-Sa	MSC876AA	2012	407.9	1.30	4.76	0.06	3.73	-0.10	-26.95	-23.73	13.32	7.5
LM-Sa	MSC877AA	2012	292.7	1.51	5.10	0.10	3.95	-0.09	-27.75	-24.23	12.64	8.3
LM-Sa	MSC878AA	2012	394.9	1.30	4.53	0.06	3.47	-0.18	-27.26	-23.81	12.56	8.1
LM-Sa	MSC879AA-ave	2012	316.0	1.33	4.78	0.04	3.70	-0.11	-27.67	-24.05	12.75	8.6

Table S2. The Hg stable isotopes and HgT concentrations for 2 dated sediment cores collected in Lake Michigan.UW ID is the unique University fo Wisconsin Madison identifier code.

	Top cm	Base cm	Mid Date year	UW ID	[HgT] ng g^{-1}	Sedimentation rate g cm^{-2} yr^{-1}	Hg accumulation rate ng cm^{-2} yr^{-1}	$\delta^{202}Hg$ ‰	$\Delta^{199}Hg$ ‰	$\Delta^{200}Hg$ ‰	$\Delta^{201}Hg$ ‰	$\Delta^{204}Hg$ ‰
North Lake Michigan, Manitou Pass, 2010	0.00	0.25	2010	UW-DRE-001	115.0	0.042	4.9	-0.76	0.20	0.07	0.16	-0.08
	0.25	0.50	2009	UW-DRE-002	112.0	0.049	5.5	-0.64	0.20	0.08	0.14	-0.12
	0.50	0.75	2008	UW-DRE-003	100.7	0.049	4.9	-0.72	0.20	0.07	0.16	-0.13
	0.75	1.00	2006	UW-DRE-004	95.5	0.049	4.7	-0.70	0.21	0.09	0.16	-0.14
	1.00	1.25	2005	UW-DRE-005	99.4	0.049	4.8	-0.68	0.22	0.09	0.14	-0.15
	1.25	1.50	2004	UW-DRE-006	92.1	0.049	4.5	-0.69	0.22	0.06	0.12	-0.11
	1.50	1.75	2003	UW-DRE-007	91.6	0.056	5.1	-0.71	0.21	0.08	0.11	-0.15
	1.75	2.00	2001	UW-DRE-008	88.3	0.062	5.5	-0.79	0.17	0.03	0.14	-0.09
	2.00	2.25	2000	UW-DRE-009	92.7	0.062	5.8	-0.76	0.19	0.05	0.14	-0.17
	2.25	2.50	1999	UW-DRE-010	103.4	0.062	6.4	-0.76	0.21	0.07	0.16	-0.05
	2.50	2.75	1997	UW-DRE-011	106.2	0.062	6.6	-0.73	0.19	0.07	0.13	-0.16
	2.75	3.00	1996	UW-DRE-012	114.1	0.062	7.1	-0.82	0.21	0.07	0.15	-0.08
	3.00	3.25	1995	UW-DRE-013	110.5	0.070	7.7	-0.75	0.24	0.06	0.19	-0.09
	3.25	3.50	1993	UW-DRE-014	109.5	0.075	8.2	-0.78	0.20	0.02	0.16	-0.18
	3.50	3.75	1992	UW-DRE-015	117.5	0.075	8.8	-0.80	0.20	0.04	0.10	-0.14
	3.75	4.00	1991	UW-DRE-016	118.1	0.075	8.9	-0.82	0.21	0.05	0.13	-0.12
	4.25	4.50	1989	UW-DRE-017	111.5	0.075	8.4	-0.77	0.24	0.06	0.13	-0.11
	4.75	5.00	1986	UW-DRE-018	117.4	0.081	9.5	-0.77	0.21	0.06	0.07	-0.05
	5.75	6.00	1982	UW-DRE-019	115.2	0.081	9.3	-0.75	0.23	0.07	0.14	-0.09
	6.75	7.00	1977	UW-DRE-020	131.3	0.084	11.0	-0.73	0.22	0.04	0.13	-0.03
South Lake Michigan, H22-1, 2009	0.25	0.50	2009.1	UW-DRE-37	64.9	0.302	19.6	-0.58	0.06	0.04	0.01	-0.06
	1.25	1.50	2007.9	UW-DRE-38	70.2	0.239	16.8	-0.61	0.06	0.03	0.07	-0.07
	2.25	2.50	2006.1	UW-DRE-39	71.6	0.242	17.3	-0.53	0.06	0.04	0.04	-0.08
	3.25	3.50	2004.3	UW-DRE-40	72.0	0.259	18.6	-0.61	0.06	0.02	0.06	-0.04
	4.25	4.50	2002.5	UW-DRE-41	72.6	0.259	18.8	-0.52	0.09	0.04	0.07	-0.03
	5.25	5.50	2000.7	UW-DRE-42	80.4	0.221	17.8	-0.57	0.13	0.07	0.10	-0.07
	6.25	6.50	1998.7	UW-DRE-43	73.5	0.221	16.2	-0.61	0.13	0.06	0.10	-0.06
	6.75	7.00	1997.6	UW-DRE-44	67.2	0.188	12.6	-0.62	0.09	0.03	0.02	-0.06
	7.50	8.00	1995.6	UW-DRE-45	75.3	0.198	14.9	-0.61	0.08	0.05	0.05	-0.03
	8.50	9.00	1993.3	UW-DRE-46	80.2	0.211	16.9	-0.57	0.10	0.04	0.06	-0.09
	10.00	10.50	1990	UW-DRE-47	78.4	0.205	16.0	-0.61	0.09	0.03	0.08	0.00
	11.00	11.50	1987.1	UW-DRE-48	79.0	0.197	15.5	-0.70	0.07	0.05	0.06	-0.05
	12.00	12.50	1984.3	UW-DRE-49	86.5	0.197	17.0	-0.78	0.08	0.05	0.05	-0.04
	13.00	13.50	1981.3	UW-DRE-50	74.6	0.212	15.8	-0.82	0.06	0.02	0.03	0.02
	14.00	14.50	1978.3	UW-DRE-51	79.4	0.212	16.8	-0.74	0.07	0.05	0.02	0.01

Table S3. The HgT stable isotope ratio triplicates quality assurance and quality control data. Relative standard deviation and standard deviation are marked as RSD and sd respectively.

USGS ID	Year	[HgT] ng/g DW	RSD	δHg202	sd	ΔHg199	sd	ΔHg200	sd	ΔHg 201	sd	ΔHg204	sd
MSC710AA	1977	490.9	8%	0.83	0.01	5.31	0.08	0.11	0.00	4.11	0.04	-0.15	0.01
MSC820AA	1998	456.2	1%	1.14	0.08	5.04	0.08	0.10	0.01	3.96	0.05	-0.17	0.03
MSC830AA	2001	389.0	9%	1.48	0.09	4.95	0.03	0.10	0.01	3.87	0.00	-0.20	0.00
MSC720AA	1978	764.6	2%	0.56	0.03	5.19	0.02	0.04	0.02	4.01	0.01	-0.06	0.03
MSC760AA	1986	573.1	2%	0.54	0.01	5.04	0.01	0.06	0.02	3.96	0.02	-0.12	0.04
MSC770AA	1989	233.2	7%	0.42	0.06	4.67	0.04	0.10	0.01	3.67	0.05	-0.08	0.02
MSC780AA	1990	310.5	8%	0.95	0.10	4.87	0.01	0.09	0.01	3.82	0.02	-0.14	0.02
MSC790AA	1991	250.7	5%	1.07	0.08	5.17	0.03	0.09	0.03	4.04	0.04	-0.15	0.03
MSC800AA	1993	287.8	4%	1.08	0.05	5.08	0.03	0.08	0.02	3.97	0.01	-0.10	0.01
MSC810AA	1996	315.5	4%	1.27	0.07	5.41	0.02	0.10	0.02	4.26	0.04	-0.18	0.01
MSC870AA	2011	273.7	5%	1.40	0.05	4.80	0.04	0.10	0.01	3.71	0.01	-0.13	0.03
MSC730AA	1979	919.7	4%	0.79	0.04	5.17	0.03	0.07	0.01	4.02	0.01	-0.14	0.04
MSC740AA	1980	175.3	7%	0.48	0.02	4.15	0.02	0.09	0.02	3.25	0.01	-0.07	0.02
IAEA407		100%	3%	0.56	0.03	1.08	0.02	0.03	0.02	0.87	0.02	-0.03	0.02

Table S4. Triplicate quality assurance data for the carbon and nitrogen isotope analyses.

	**δ13C**	**sd**	**δ15N**	**sd**
MSC820AA	-28.4	0.1	14.6	0.1
MSC850AA	-27.3	0.1	14.3	0.1
MSC879AA	-27.7	0.0	12.8	0.2
MSC870AA	-27.2	0.2	13.9	0.2
MSC830AA	-28.9	0.2	14.7	0.2
MSC860AA	-27.8	0.1	15.1	0.3
MSC841AA	-29.2	0.0	14.2	0.4

Table S5. Secchi depths for Lake Michigan. Raw data including locations may be found on the U.S. EPA GLNPO, Great Lakes Environmental Database (cdx.epa.gov).

Season	Year	2-yr av	sd	Max	Min	Season	Year	2-yr av	sd	Max	Min
Spring	1983	9.6	2.1	11.6	7.5	Spring	2000	7.7	1.7	9.4	6.0
Summer	1983.5	9.8	1.8	11.7	8.0	Summer	2000.5	8.2	1.1	9.3	7.1
Spring	1984	9.8	2.6	12.4	7.2	Spring	2001	8.3	2.1	10.3	6.2
Summer	1984.5	9.9	3.1	13.1	6.8	Summer	2001.5	8.9	1.1	9.9	7.8
Spring	1985	10.4	1.9	12.4	8.5	Summer	2002.5	9.4	1.3	10.7	8.1
Summer	1985.5	10.3	3.0	13.3	7.4	Spring	2003	10.2	1.0	11.2	9.2
Spring	1986	9.9	2.2	12.1	7.7	Summer	2003.5	10.6	2.6	13.1	8.0
Summer	1986.5	9.5	3.8	13.3	5.8	Spring	2004	11.5	2.5	13.9	9.0
Spring	1987	9.1	2.6	11.8	6.5	Summer	2004.5	11.9	1.8	13.7	10.1
Summer	1987.5	9.4	1.3	10.7	8.0	Spring	2005	12.5	2.2	14.7	10.4
Spring	1988	8.8	1.3	10.1	7.5	Summer	2005.5	12.9	2.0	15.0	10.9
Summer	1988.5	8.5	1.5	9.9	7.0	Spring	2006	13.4	1.6	15.0	11.9
Spring	1989	8.3	1.4	9.8	6.9	Summer	2006.5	13.1	2.2	15.3	10.9
Summer	1989.5	8.8	2.0	10.8	6.9	Spring	2007	13.2	4.5	17.7	8.7
Spring	1990	8.5	2.3	10.7	6.2	Summer	2007.5	13.7	2.0	15.7	11.7
Summer	1990.5	8.4	2.3	10.6	6.1	Spring	2008	13.9	1.6	15.5	12.2
Spring	1991	7.5	1.9	9.4	5.6	Summer	2008.5	13.6	4.3	17.9	9.3
Summer	1991.5	7.8	1.1	8.9	6.6	Spring	2009	13.6	1.7	15.3	11.9
Spring	1992	7.3	2.5	9.9	4.8	Summer	2009.5	13.5	2.1	15.6	11.4
Summer	1992.5	7.2	1.4	8.5	5.8	Spring	2010	13.3	3.9	17.2	9.5
Spring	1993	6.9	2.4	9.4	4.5	Summer	2010.5	13.4	2.6	16.0	10.8
Summer	1993.5	7.1	1.0	8.1	6.2	Spring	2011	13.2	2.8	16.0	10.4
Spring	1994	7.1	1.2	8.3	6.0	Summer	2011.5	13.7	2.7	16.3	11.0
Summer	1994.5	7.6	1.6	9.2	6.0	Spring	2012	14.0	3.7	17.7	10.3
Spring	1995	7.8	1.1	8.9	6.7	Summer	2012.5	14.7	2.5	17.1	12.2
Summer	1995.5	8.5	1.5	10.0	7.1	Spring	2013	14.4	1.4	15.9	13.0
Spring	1996	8.4		8.4	8.4	Summer	2013.5	15.4	2.2	17.6	13.2
Summer	1996.5	8.4	1.9	10.3	6.5	Spring	2014	15.5	2.0	17.5	13.5
Spring	1997	8.2	2.3	10.4	5.9	Summer	2014.5	15.9	2.2	18.1	13.7
Summer	1997.5	8.3	1.4	9.7	6.9	Spring	2015	15.7	2.9	18.6	12.9
Spring	1998	7.9	3.0	10.9	4.9	Summer	2015.5	16.6	1.8	18.4	14.8
Summer	1998.5	7.8	2.2	10.0	5.5	Spring	2016	16.3	5.1	21.4	11.3
Spring	1999	7.6	1.9	9.5	5.6	Summer	2016.5	17.0	3.2	20.3	13.8
Summer	1999.5	7.9	1.0	8.9	7.0	Spring	2017	16.8	4.7	21.5	12.1

References

1. Hoffman, J. C., Sierszen, M. E. & Cotter, A. M. Fish tissue lipid-C:N relationships for correcting $\delta^{13}C$ values and estimating lipid content in aquatic food-web studies. *Rapid Commun. Mass Spectrom.* 29, 2069–2077 (2015).
2. Turschak, B. A. Bunnell, D.; Czesny, S.; Höök, T. O.; Janssen, J.; Warner, D.; Bootsma, H. A., Nearshore energy subsidies support Lake Michigan fishes and invertebrates following major changes in food web structure. *Ecology* 95, 1243–1252 (2014).
3. Fry, B. Stable isotopic indicators of habitat use by Mississippi River fish. *J. North Am. Benthol. Soc.* 21, 676–685 (2002).
4. Bergquist, B. A. & Blum, J. D. Mass-dependent and -independent fractionation of Hg isotopes by photoreduction in aquatic systems. Science 318, 417–420 (2007).
5. Point, D. et al. Methylmercury photodegradation influenced by sea-ice cover in Arctic marine ecosystems. *Nat. Geosci.* 4, 188–194 (2011).
6. Lepak, Ryan F., Janssen, S. E.; Yin, R.; Krabbenhoft, D. P.; Ogorek, J. M.; DeWild, J. F.; Tate, M. T.; Holsen, T. M.; Hurley, J. P., "Factors Affecting Mercury Stable Isotopic Distribution in Piscivorous Fish of the Laurentian Great Lakes." *Environmental Science & Technology* 52.5 (2018): 2768-2776.
7. Rose, C. H., Ghosh, S., Blum, J. D. & Bergquist, B. A. Effects of ultraviolet radiation on mercury isotope fractionation during photo-reduction for inorganic and organic mercury species. *Chem. Geol.* 405, 102–111 (2015).
